# Dynamics of hinged wings in strong upward gusts

**DOI:** 10.1098/rsos.221607

**Published:** 2023-05-10

**Authors:** Jonathan P. J. Stevenson, Jorn A. Cheney, James R. Usherwood, Richard J. Bomphrey, Shane P. Windsor

**Affiliations:** ^1^ Department of Aerospace Engineering, University of Bristol, Bristol BS8 1TR, UK; ^2^ Structure and Motion Laboratory, Royal Veterinary College, Hatfield AL9 7TA, UK

**Keywords:** gust rejection, bird flight, hinged wings, centre of percussion, suspension system, soft stall

## Abstract

A bird's wings are articulated to its body via highly mobile shoulder joints. The joints confer an impressive range of motion, enabling the wings to make broad, sweeping movements that can modulate quite dramatically the production of aerodynamic load. This is enormously useful in challenging flight environments, especially the gusty, turbulent layers of the lower atmosphere. In this study, we develop a dynamics model to examine how a bird-scale gliding aircraft can use wing-root hinges (analogous to avian shoulder joints) to reject the initial impact of a strong upward gust. The idea requires that the spanwise centre of pressure and the centre of percussion of the hinged wing start, and stay, in good initial alignment (the centre of percussion here is related to the idea of a ‘sweet spot’ on a bat, as in cricket or baseball). We propose a method for achieving this rejection passively, for which the essential ingredients are (i) appropriate lift and mass distributions; (ii) hinges under constant initial torque; and (iii) a wing whose sections stall softly. When configured correctly, the gusted wings will first pivot on their hinges without disturbing the fuselage of the aircraft, affording time for other corrective actions to engage. We expect this system to enhance the control of aircraft that fly in gusty conditions.

## 1. Introduction

Flight in the low atmosphere (below 1000 m) is challenging and often dangerous. Flyers must not only steer clear of obstacles, terrain and other airborne objects, but also contend with strong winds and gusty flows [1,2]. Birds are light and fly relatively slowly; they become increasingly vulnerable as the windspeed rises [3]. Nevertheless, many of them manage to fly with remarkable agility and control in this realm of the atmosphere, with their wings and tails constantly tilting and flexing (e.g. [4]) in response to the unsteady wind. Designers are now seeking to harness the mechanical principles of avian wings to enhance the control of novel aircraft [5,6].

In this study, we focus on a specific idea: the utility of wing-root hinges for gust rejection on aircraft, particularly small unmanned aerial vehicles. The work was motivated by a laboratory experiment in which a gliding barn owl responded to strong upward gusts (with a magnitude of 40–70% of the flight speed and a width greater than the wingspan) by immediate rotation of its wings around the shoulder joints [7]. The motion of the wing masses effectively absorbed the sudden impulse from the extra aerodynamic load, allowing the torso and head, together equivalent to a fuselage, to maintain a smooth flight trajectory. Cheney *et al.* [7] named this effect ‘inertial rejection’; it relies purely on the displacement of wing inertia and does not require active control.

Inertial rejection can be enhanced by exploiting an intrinsic property of hinged wings and, for that matter, all rigid (or near-rigid) pivoting masses: the *centre of percussion*. It is that point on the mass at which a sudden transverse load does not transmit any immediate reaction force through the pivot. In other words, the pivot will not ‘feel’ anything in the transverse direction. Players of bat-and-ball sports, particularly cricket and baseball, will have practical familiarity with the centre of percussion, as it is closely related to the so-called *sweet spot*—that special zone on a bat where a ball can be struck without causing unpleasant jarring of the hands [8,9]. In general, the centre of percussion *P* of a hinged object, of mass *m*, is located at a distance (electronic supplementary material part 1, §6)

1.1 $$P=\frac{I}{md},$$

from the pivot point. Distance *d* is that from the hinge to the centre of mass (CoM) of the object, and *I* is the corresponding mass moment of inertia about the hinge.

As such, hinged wings can perform a similar role to suspension systems on terrestrial vehicles. Sabins [10] patented an early imagining of a suspension system for light aircraft, in which each (rigid) wing was hinged to the fuselage on a root pin joint and supported on a shock-absorbing strut. The wings were to deflect up or down in response to changing air loads in flight, and to shocks during landing, with the aim of reducing structural stresses and enhancing passenger comfort. More recently, studies have suggested that wing-root hinges can improve the gust response of small-scale aircraft [11,12] and may even provide opportunities for tuning flight stability and performance [13,14]. To our knowledge, though, gust-rejecting hinged wings have not yet seen mainstream application on commercial aircraft at any scale, nor has the centre of percussion been used to enhance any of the designs that do exist.

In this study, we develop a dynamics model to examine how the action of a wing-root hinge can mitigate the initial impact of an upward gust (hereafter *upgust*) on the fuselage of a small gliding aircraft. The gust is wider than the wingspan of the flyer, as in the owl experiment of Cheney *et al*. [7]. Having explored the nature of the centre of percussion and its basic role in the transmission of load from the hinged wings to the fuselage, we propose a method to exploit the mechanics for immediate passive gust rejection. We call this the *percussion effect*. Altogether, the results inform the design of novel wing suspension systems for aircraft that require smoother flight, e.g. those carrying fragile payloads, cameras or sensors in difficult conditions.

## 2. Methods

**Figure 1.**
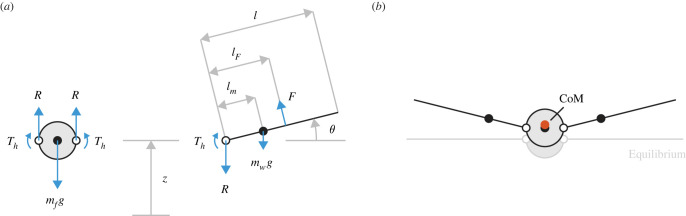
The modelled system. The depicted motion is illustrative; the arrow lengths are only intended to give a qualitative sense of the relative magnitude of the loads. (*a*) Free-body diagrams for the fuselage (left) and wing (right) masses, horizontal forces omitted (they do not affect the vertical dynamics). The fuselage can translate vertically, while each wing can hinge about its root end on a pin joint (open circle). The fuselage is regarded as dimensionless, and the aerodynamic force upon it is neglected. (*b*) Motion occurs when extra vertical force on the wings pushes the system away from equilibrium. The CoM of the overall system (filled orange circle) lies, by definition, between those of the wings and that of the fuselage (filled black circles).

The modelled system is a mechanical analogue for a bird, consisting of a central fuselage (torso) mass and two rigid wing beams (figure 1). Each wing root is hinged to the fuselage on a pin joint. The fuselage is free to translate along a vertical line, while the wings can rotate on their hinges in symmetry, i.e. the wings are identical and their motion is mirrored about the vertical centreline (this representation is based on the *initial* kinematic response of the owl to an upgust, in which the wings rotated symmetrically about the shoulders without significant bending or twist). As such, the system has two degrees of freedom; we choose fuselage height *z* and wing angle *θ* (from the horizontal) as the most convenient generalized coordinates.

At first, the system travels in level equilibrium flight (lift equals system weight, an approximation for the glide condition) with constant forward flight speed *U*. The initial relative wind is therefore horizontal, also with magnitude *U*. The system then encounters a strong upgust (whose width far exceeds the wingspan). This produces extra lift on the wings and impels them upwards, eventually causing the fuselage to translate vertically as it receives load via the hinges. We seek to understand these vertical motions, particularly at the level of the fuselage.

In this reduced-order model, we neglect both pitch and drag. The system does not possess a degree of freedom in pitch; we assume that any motion about the lateral axis is controlled separately. Nevertheless, note that: (i) at low angles of attack, at least, the system pitching moment should not change significantly, provided the hinge is located near the chordwise aerodynamic centre of the wing, and (ii) the system has no tail, so there is no other source of pitching moments. Further, for the changes in angles of attack we consider, the upward component of wing drag is small enough not to affect the motion of the system.

Figure 1 shows free-body diagrams for the fuselage and a single wing, all horizontal forces omitted. The relevant equations of motion for this system are (see electronic supplementary material part 1, §1)

2.1 $$M\;\ddot{z}+2m_{w}l_{m}(\ddot{\theta }\cos\theta -{\dot{\theta }}^{2}\sin\theta )+Mg=2F\cos\theta ,$$

which derives from the balance of forces on the overall CoM, and

2.2 $$I_{h}\ddot{\theta }+m_{w}l_{m}\ddot{z}\cos\theta +\;T_{h}+m_{w}gl_{m}\cos\theta =Fl_{F},$$

which is the sum of moments on each wing about its hinge. Table 1 shows the nomenclature of system parameters. Note that CoM distance *l_m_* and mass moment of inertia *I_h_* are dictated by the wing mass distribution, as discussed shortly. On the right-hand side of the equations, *F* is the component of wing lift in the plane of motion and acts at distance *l_F_* from the hinge.

**Table 1.** Properties and inputs of the modelled system.

symbol	description	value	unit
*g*	magnitude of gravitational acceleration	9.81	m s^−2^
*m_f_*	mass of fuselage	0.25	kg
*m_w_*	mass of single wing	0.025	kg
*M*	mass of overall system (= *m_f_* + 2*m_w_*)	0.3	kg
*μ_f_*	mass fraction of the fuselage (= *m_f_*/*M*)	0.8	—
*μ_w_*	mass fraction of the wings (= 2*m_w_*/*M*)	0.2^a^	—
*c*	chord length of wing section	0.15	m
*l*	spanwise length of wing	0.4	m
*l_m_*	CoM of wing from hinge	0.133	m
*I_h_*	mass moment of inertia of wing about hinge	6.67×10^-4^	kg m^2^
*P*	centre of percussion of wing from hinge	0.2	M
*k_t_*	torsional hinge stiffness	0^a^	N m rad^−1^
*ρ*	air density	1.2	kg m^−3^
*U*	forward flight speed (= relative wind)	8.0	m s^−1^

^a^These are varied later.

We model the ‘total’ torque *T_h_* at each hinge as the sum of static (*T_h_*_0_) and dynamic parts (Δ*T_h_*) (we give all static quantities a subscript 0, and dynamic quantities a leading Δ). Thus, *T_h_* = *T_h_*_0_ + Δ*T_h_*. The static torque is simply the amount required to keep each wing in its horizontal equilibrium position for ordinary level flight. It is found by combining equations (2.1) and (2.2) at equilibrium:

2.3 $$T_{h0}=\frac{Mg}{2}l_{F0}-m_{w}gl_{m}.$$

In this model, it is restorative in sense, i.e. acting to pull each wing downward (note that *l_m_* < *l_F_*_0_ and *m_w_* ≪ *M*, cf*.* Table 1). This is also true for birds [15]. Static torque is necessarily present in all of our modelled cases, and the hinge is presumed to be able to supply the precise amount. The dynamic torque Δ*T_h_* is the change about the static value. It arises when the wing deviates from a horizontal position. In §3.5, we consider the effect of a hinge whose dynamic torque has the behaviour of a linear torsional spring (Δ*T_h_* = *k_t_θ*, where *k_t_* is the stiffness). As such, our modelling of the hinge is abstract; we leave the detailed mechanical design to another study.

In addition to its own weight, the fuselage experiences a vertical reaction force from the wing at each hinge. We denote this reaction as *R* (figure 1). Its force complement acts on the wing, with equal magnitude but opposite sense. The equation of motion for the fuselage is therefore

2.4 $$R=\frac{m_{f}g+m_{f}\ddot{z}}{2}.$$

Note that *R* has both static (*R*_0_ = *m_f_g*/2) and dynamic ($\Delta R=m_{f}\ddot{z}/2$) parts. The dynamic part determines the response of the fuselage to the gust.

Frequent comparison is made in later sections to a system with ‘fixed’ wings, i.e. a rigid-wing aircraft without hinges. Its equation of motion is

2.5 $$M\ddot{z}+Mg=2F.$$

In this case, the wings and fuselage move in tandem. The point of application of force *F* therefore does not matter.

### 2.1. Linearization

The equations of motion for the hinged system are nonlinear, second-order and coupled by the generalized coordinates. We now linearize them about *θ* = 0 to simplify the problem for clearer insight on the basic mechanics. This does limit the results to |θ|≲20 degrees (when the small-angle approximation cos*θ* ≃ 1 passes 5% error), but the percussion effect is expected to be most applicable at low angles anyway.

Using the small-angle approximation (cos*θ* ≃ 1) and the fact that the initial angular wing velocity is small, especially when squared (${\dot{\theta }}^{2}≃0$), we find the linearized equations of motion

2.6 $$M\ddot{z}+2m_{w}l_{m}\ddot{\theta }+Mg=2F$$

and

2.7 $$I_{h}\ddot{\theta }+m_{w}l_{m}\ddot{z}+T_{h}+m_{w}gl_{m}=Fl_{F}.$$

One further simplification—that the total load be regarded as the sum of static and dynamic parts—allows equilibrium loads to be subtracted from these equations. Therefore (see electronic supplementary material part 1, §2),

2.8 $$M\ddot{z}+2m_{w}l_{m}\ddot{\theta }=2\Delta F$$

and

2.9 $$I_{h}\ddot{\theta }+m_{w}l_{m}\ddot{z}+\Delta T_{h}=\Delta Fl_{\Delta F}.$$

Force *F* (hence Δ*F*) now effectively points upwards at all times.

### 2.2. System parameters

The size and overall mass of the system are based on the owl in Cheney *et al*. [7]. Some parameters have been rounded for convenience (table 1). As such, we are strongly inspired by the dynamics of the owl, but do not intend to mimic all its aeromechanical complexities.

For the spanwise wing mass distribution, we choose a linear function. This is simply an approximation for the wings of birds (see van den Berg & Rayner [16], for example) and aircraft alike. For a wing of length *l*, it has the form (see electronic supplementary material part 1, §3)

2.10 $$m_{w}^{\prime }=-\frac{2m_{w}}{l}\left(\frac{y}{l}-1\right),$$

where *y* is the spanwise coordinate, running from root to tip. The centre of percussion *P* of this mass distribution lies at *l*/2, which, as discussed shortly, aligns perfectly with the centre of pressure of our chosen equilibrium lift distribution. This case of perfect alignment interests us here because it may provide the largest potential gust rejection benefit (for an interesting comparison, note that Cheney *et al*. [7] found that equilibrium alignment is *almost* true for the barn owl—within just 0.067*l*, where *l* is the wing length, as defined here). Of course, alignment can be achieved with other lift-mass combinations; rectangular lift and linear mass are just useful conveniences, with the latter also furnishing exact solutions for the CoM and radius of gyration (see electronic supplementary material part 1, §3 for more information on the spanwise location of these points relative to *P*), as well as the mass moment of inertia.

### 2.3. Aerodynamics formulation

As mentioned, *F* is the aerodynamic force on each wing in the (vertical) plane of motion. We implement a quasi-steady, blade-element formulation for *F*, the essential results of which are presented here. This formulation should be regarded as an adjunct to the main model just described—one possible prescription for the forcing terms on the right-hand side of the equations. Indeed, the percussion effect is purely mechanical and works properly whether the perturbing force is aerodynamic or not. In §3.1, we actually demonstrate the effect using point force alone.

Our aerodynamics formulation includes several assumptions:

(i) The wing planform is rectangular and untwisted. (That it has a linear mass distribution is explained by a hypothetical variation in material density.)
(ii) The flow is two dimensional. (Note that the effect of spanwise flow lessens with decreasing *θ*.)
(iii) All wing sections are symmetrical about the chord line and have identical aerodynamic properties.
(iv) Aeroelastic wing flexure is absent.
(v) The effect of apparent air mass is negligible.
(vi) Aerodynamic force acts in a quasi-steady manner.

With respect to point (vi), we recognize that aerodynamic force technically takes time to ‘build up’ in response to gusts and other wing motions [17,18]. As such, the force depends on the time-history of the flow. We checked this effect using classical unsteady theory, viz. Küssner and Wagner lift functions, before proceeding (see electronic supplementary material part 1, §5) and found that: (i) the unsteady system response is similar in shape and character to the quasi-steady result; and (ii) ‘build-up’ introduces a time delay. We therefore decided, for simplicity and directness in the modelling, to use the quasi-steady approach throughout, acknowledging that comparisons between our cases would be more important than the absolute timings of any single one. This quasi-steady assumption is also quite common in the analysis of flapping wings (e.g. [19]), whose dynamics are reminiscent of the present model. The formulation now follows (see electronic supplementary material part 1, §4 for details).

The upward projection of *F* (i.e. *F*cos*θ*) determines the vertical motion of the system, and, in our linearization of *θ*, is approximately equivalent to *F* itself. All subsequent references to *F* therefore refer to this vertical projection. Of course, *F* is actually the integral resultant of the spanwise lift distribution, which is given by (when linearized)

2.11 $$F^{\prime }(y)=qcc_{L}(\alpha_{0}+\Delta \alpha ),$$

where *q* = *ρU*^2^/2 is the dynamic pressure and *c* is the (constant) chord length of the wing. Recall that all wing sections are identical and have the same lift curve. The lift coefficient distribution *c_L_* therefore depends only upon: (i) the static angle of attack (AoA) *α*_0_, necessary for weight support at equilibrium, and (ii) the dynamic AoA increment Δ*α*(*y*), which embodies any changes brought about by the gust and/or motion of the system. The tested gust is in fact moderate enough to permit linearization of Δ*α*; we therefore find that (see electronic supplementary material part 1, §4)

2.12 $$\Delta \alpha (y)≃\frac{v_{g}-\dot{z}-y\dot{\theta }}{U},$$

where *v_g_* is the gust velocity, introduced shortly. The total force on each wing is then

2.13 $$F=qc\int_{0}^{l}c_{L}(\alpha_{0}+\Delta \alpha )\;dy,$$

with a corresponding moment about the hinge axis given by

2.14 $$M_{F}=qc\int_{0}^{l}yc_{L}(\alpha_{0}+\Delta \alpha )\;dy.$$

In our calculations, we use discretized forms of these integrals with 50 spanwise points along each wing.

Quotient *M_F_*/*F* = *l_F_* defines the spanwise point of action or *centre of pressure* of the force *F*. At equilibrium, of course, there is no gust or wing motion and Δ*α* = 0 everywhere; the force distribution *F*^′^ is rectangular, and its centre of pressure (resultant) *F*_0_ lies halfway along the span of the wing (*l*/2). This puts it into equilibrium alignment with the centre of percussion. We return to this point again in §3.2.

Velocity *v_g_* is the upgust intensity along the direction of flight. We use the standard ‘1 – cosine’ profile from the FAA airworthiness regulations [20],

2.15 $$v_{g}(t)=\frac{v_{gA}}{2}\left(1-\cos2\pi \frac{Ut}{L_{g}}\right),$$

where *v_gA_* is the peak intensity, and *L_g_* the physical length, of the gust (table 2). The gust is homogeneous in the spanwise direction; it can be imagined as an unlimited spanwise extrusion of this cosine profile. In this study, we test a nominal peak gust velocity of 30% forward speed, or 0.3*U* (by nominal, we mean that the Küssner function has not been applied). Such a gust would seriously challenge the flight of most animals and small aircraft, but is still moderate enough not to undermine the assumption of linearity during the initial response of the wing.

**Table 2.** Properties of the vertical gust, also based on Cheney *et al*. [7].

symbol	description	value	unit
*v_gA_*	peak gust velocity	2.4	m s^−1^
*L_g_*	gust length along flight direction	1.4	m

Temporal solution of the equations of motion was carried out in MATLAB® (MathWorks, Massachusetts) using the ODE45 Runge–Kutta solver. At each time point, the aerodynamic loads were computed from the current state variables before being passed to the equations for solution of the next iteration. The algorithm automatically adjusted the size of the time step according to local gradients in the solution. Still, for robustness, we specified a *maximum* time step of one-sixth of the gust duration (*L_g_*/6*U* or approx. 30 ms). This prevented the adaptive solver from taking an overly large step, ensuring, at the very least, three solution points per side of the gust (we did test, for the simulation conditions in figure 3, whether the solution result was sensitive to this maximum time step, particularly when made shorter, but it was not). Once finished, ODE45 returned a solution sampled at the requested time points—in this case, every 5 ms.

## 3. Results

First, we develop the basic mechanics of the centre of percussion for general point force (§3.1). We then extend the analysis to distributed aerodynamic loading, considering two lift curves: one with constant slope (§3.2) and another with ‘soft-stall’ behaviour (§3.3). We also explore the effect of relatively heavy wings (§3.4). Finally, we introduce dynamic hinge torque (§3.5) via linear torsional stiffness.

### 3.1. Basic mechanics

Hinges allow the wings to rotate in response to external impulses, including gusts. Consider first a system with hinges that each produce a constant torque, equal only to the static value (*T_h_* = *T_h_*_0_, Δ*T_h_* = 0). The system therefore supports level flight *exactly*. If either wing experiences a transverse perturbing force Δ*F*, an associated reaction increment Δ*R* may develop, or be *transmitted*, to the fuselage via the corresponding hinge (we omit ‘increment’ hereon). The underlying mechanics are embodied by the equations of motion; from their linearized forms, we can derive a concise expression that links Δ*F* to Δ*R* (see electronic supplementary material part 1, §6), given by

3.1 $$\Delta R=\Delta F\frac{\left(P-l_{\Delta F}\right)}{C},$$

where *l*_Δ_*_F_* is the distance from the hinge to the force Δ*F*, *P* is the position of the centre of percussion on the wing, and *C* = *P* − (*μ_w_*/*μ_f_*)(*l_m_* − *P*) is a positive constant. The formula works best at moderate wing angles (|θ|≲20 degrees, at which the approximation cos*θ* ≃ 1 crosses 5% error) and therefore applies during the crucial early moments of the response.

According to this expression, it is the position and magnitude of force Δ*F* that govern the initial transmission of reaction Δ*R* to the fuselage; static loads do not matter. Assuming, for now, that the acting point force is constant, there are three possible scenarios: (i) inboard or ‘armpit’ loading, in which Δ*F* acts inside *P* (immediate upward reaction develops on the fuselage, with a magnitude that depends on the degree of misalignment with *P*); (ii) outboard loading, in which Δ*F* acts outside *P* (reaction now develops in the downward direction, with a magnitude that again depends on the misalignment); or (iii) aligned loading, in which Δ*F* acts *at P*. The bracketed term in equation (3.1) vanishes so no immediate reaction develops. This *percussion effect* works regardless of the magnitude of Δ*F*. Note also that if the reaction is nullified, so too is the rolling moment it would otherwise apply to the fuselage (particularly important when the wings are loaded asymmetrically and these moments do not balance out).

**Figure 2.**
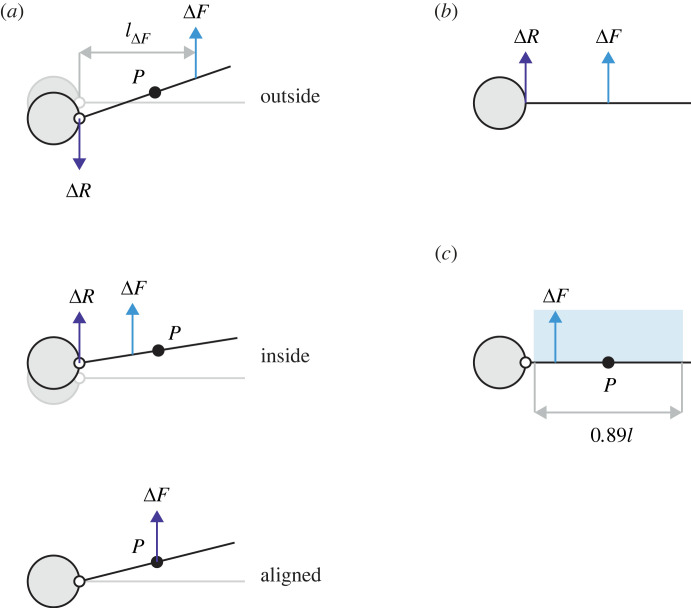
Pseudo free-body diagrams of load transmission from wing to fuselage. Hinge torque is constant and equal to the static value. (*a*) Linearized dynamics of the hinged wing. The direction of the fuselage reaction increment Δ*R* varies with the spanwise location of the applied force Δ*F*. Force through *P* produces no immediate reaction. (*b*) Dynamics of the fixed wing. Δ*R* acts in the same direction as Δ*F* with magnitude Δ*R* = *μ_f_*Δ*F*. Note that Δ*R* < Δ*F* because *μ_f_* < 1, i.e. some of the applied force is always required to accelerate the wing mass. (*c*) A hinged wing with a linear mass distribution transmits less reaction to the fuselage than a fixed wing, provided Δ*F* acts within the wide, symmetrical interval (blue, to scale) centred on *P*.

For the case of fixed wings, Δ*R* = *μ_f_*Δ*F* (see electronic supplementary material part 1, §6). Analytical comparison between the fixed and hinged cases (see electronic supplementary material part 1, §7) reveals that the fuselage experiences less absolute reaction in the latter, provided Δ*F* acts within a specific interval on the wing: *P* ± (*P* − *μ_w_l_m_*). This interval is symmetrical about *P* and its width depends on the wing mass distribution. For the linear distribution, it is quite wide, covering the central approximately 89% of the wing length. The simple process of hinging therefore modulates the transmission of reaction to the fuselage for a wide range of loading points, while optimal tuning (Δ*F* at *P*) eliminates it altogether. Figure 2 provides a complete summary.

Note that force Δ*F* (2Δ*F* with two wings) also applies an upward impulse to the system *as a whole*, and unless this is somehow countered by opposing action, the system will drift away from equilibrium indefinitely. Fortunately, aerodynamic damping prevents this in practice, but alone this is a relatively slow process. Proper control authority demands something much faster. This is an important point, which is revisited later when we discuss *aerodynamic rejection*.

### 3.2. Upgust response

In real gusts, the perturbing force Δ*F* is the integral resultant of the extra, distributed aerodynamic load that develops across each wing. To realize the percussion effect, then, we must aim to put the centre of pressure of this force at *P*. One possibility is that we align the centres of pressure and percussion at equilibrium (as we have) and then strive to *keep them together* as the wing rotates under the action of the gust. We consider one possible method shortly; first, we develop some analytical prerequisites. Hinge torque will remain constant at the static value (*T_h_* = *T_h_*_0_, Δ*T_h_* = 0) until specified otherwise.

At equilibrium, the force distribution across each wing is rectangular and constant. All wing sections operate at the same point on the lift curve. Consider first that each wing section has the same, symmetrical *linear* lift curve (LLC), or

3.2 $$c_{L}=\frac{dc_{L}}{d\alpha }\alpha ,$$

where d*c_L_*/d*α* is the (constant) slope. As such, lift varies in proportion to the AoA, and there is no stall in either direction. This is the usual choice when modelling minor gusts [18] and for the description of the extended linear region in dynamic stall [21]. When first encountered by the aircraft, the upgust begins to incline the relative wind vector from its equilibrium orientation, pushing the AoA of all wing sections up the lift curve. This occurs uniformly across the wing at first, scaling the equilibrium lift distribution such that its centroid, or centre of pressure, is momentarily preserved. As the gust force builds up, the wing is duly impelled to rotate upwards. This, in turn, induces a distribution of relative downward flow *v_y_* (hereafter *relative down-flow*) across the wing, most strongly at the tip (where linear wing speed is highest). The relative down-flow opposes the gust velocity *v_g_* and acts to temper the rising AoA Δ*α*. At any spanwise station *y*, this may be expressed with equation (2.12) as

3.3 $$\Delta \alpha ≃\frac{v_{g}-(\dot{z}+y\dot{\theta })}{U}=\frac{v_{g}-v_{y}}{U},$$

where *v_y_* is the local relative down-flow at distance *y* from the hinge, equal and opposite to the absolute vertical speed of the wing section at that location ($≃\dot{z}+y\dot{\theta }$). The presence of this relative down-flow gradient means that the spanwise force distribution along each wing becomes skewed. The centre of pressure *l_F_* therefore drifts inboard from its equilibrium position, away from the centre of percussion. The perturbing force drifts, too, and equation (3.1) promises that reaction on the fuselage will develop.

**Figure 3.**
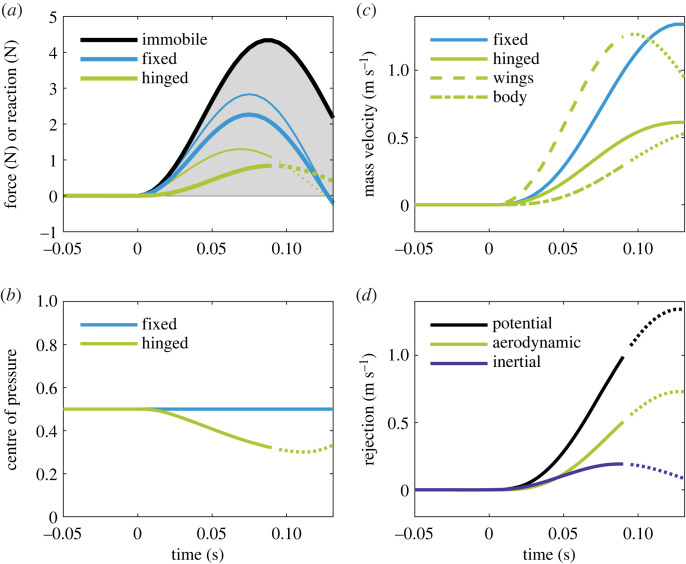
Gust response of the hinged, fixed and immobile systems with an LLC in the 30% gust. Plot lines become dotted at the instant the wing angle crosses 20 degrees (approximate onset of nonlinearity). (*a*) External force Δ*F* = *F* − *F*_0_ (thinner lines) on each wing versus dynamic fuselage reaction Δ*R* (thicker lines). For each case in turn, the reaction increment is $\Delta R=m_{f}\ddot{z}/2$ (hinged), Δ*R* = *μ_f_*Δ*F* (fixed) and Δ*R* = Δ*F* (immobile). The gust velocity profile (shaded grey) is shown for reference. (*b*) The normalized centre of pressure (*l_F_*/*l*) on each wing. On the hinged wing, it moves inboard at first, departing from *P*. (*c*) Vertical system velocity $\dot{G}$ of the hinged (plus component masses, broken lines) and fixed wing systems. The ascending wings control fuselage motion. (*d*) Rejection terms for the hinged system. The potential line indicates the maximum achievable rejection, i.e. the amount necessary to keep the fuselage perfectly level.

Figure 3 shows the impact of the tested gust (table 2) on three systems: (i) entirely immobile, in which the wings and fuselage are both clamped in their equilibrium positions throughout; (ii) fixed wing; and (iii) hinged wing. Comparison between (i) and (ii) reveals the tempering effect of relative down-flow on the applied force (figure 3*a*). The hinged wing experiences this to an even larger extent, being lightweight and better able to retreat from the gust than the fixed system (whose *full* inertia must be overcome for wing motion to begin).

The transmission of reaction force is successfully delayed in the hinged case. As the centre of pressure drifts inboard under the action of the relative down-flow gradient (figure 3*b*), though, alignment with the centre of percussion is progressively lost and fuselage reaction soon develops. The fixed wing, by contrast, transmits load instantly because its fuselage reaction simply scales directly with the external force (Δ*R* = *μ_f_*Δ*F* applies).

Cheney *et al*. [7] coined the term ‘inertial rejection’ for the idea that moving wing mass acts to stabilize the fuselage. It offers a complimentary perspective for occasions when forces are unknown or immeasurable—in a kinematics experiment like theirs, for example. Formally, they defined inertial rejection as the vertical velocity difference between the system CoM and the fuselage, i.e. as the degree of relative fuselage motion or ‘activity’ produced by the hinged wings (see electronic supplementary material part 2, §8). Inertial rejection functions best when the fuselage is made to shift by *just* the amount necessary to offset the perturbed system CoM, and this only happens when the gust force acts squarely through the centre of percussion. As such, ‘perfect’ inertial rejection *is* the percussion effect. Cheney *et al*. [7] also introduced the term ‘aerodynamic rejection’ for the difference between the external force upon (or motion of) the hinged and fixed wing systems. In other words, it is a comparison of the aerodynamic control between the two (note that Cheney *et al.* [7] used vertical velocity from their kinematics data, equivalent to impulse). Altogether, the idea is that inertial rejection lessens the *internal* reaction on the fuselage, while aerodynamic rejection modulates the *external* force on the whole system (via wing morphing or other mechanisms, including relative down-flow). The overall motion is thereby controlled. Indeed, without a reduction in the external force to complement and/or follow inertial rejection, the system would need to rely solely on natural damping from relative down-flow to arrest the acquired motion.

Figure 3*c* shows how the wings accelerate upwards to absorb the initial brunt of the gust. Inertial rejection is positive but by no means optimal (figure 3*d*). Aerodynamic rejection is comparable in magnitude at first, only increasing once the effect of relative down-flow on the hinged wing exceeds that on the fixed, as described. This does not match qualitatively the owl data in Cheney *et al*. [7], for which inertial rejection is dominant initially.

### 3.3. Rejection by soft stall

Broadly speaking, low-speed aerofoils stall in one of three ways [22]; two of these precipitate from the leading edge, and the other from the trailing edge. The latter, trailing-edge type is often associated with thicker aerofoils and tends to produce a lift curve whose linear region ends without a sudden drop in lift—so-called ‘soft’ stall [23,24]. This contrasts with ‘hard’ stall, in which lift falls off abruptly, and often unfavourably, after the linear region. Some soft-stall aerofoils are designed to produce lift curves that reach a maximum and stay there [25]. Their curves plateau, or move onto a region of shallow decline, where lift is largely insensitive to the AoA. It transpires that soft-stall lift curves could enhance the percussion effect by passive means.

**Figure 4.**
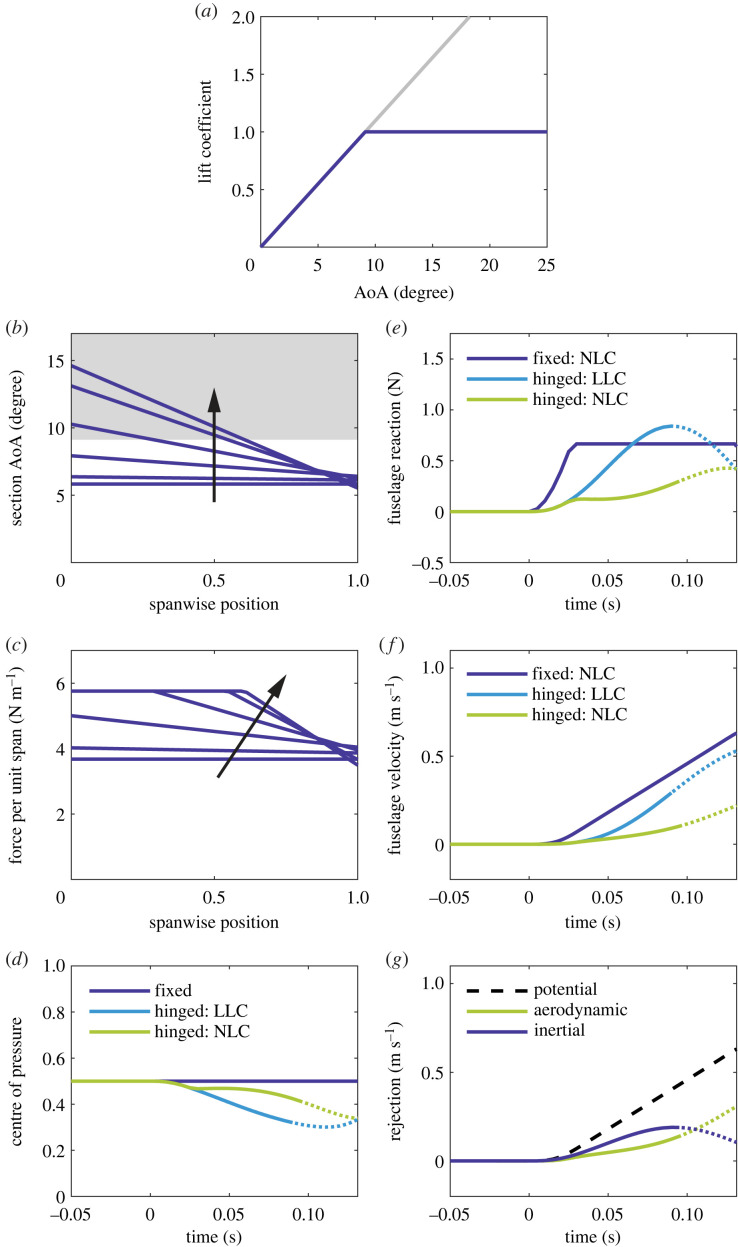
Gust response of the hinged and fixed systems with an LLC/NLC in the 30% gust. Plot lines become dotted at the instant the wing angle crosses 20 degrees (the approximate onset of nonlinearity). (*a*) The NLC lift coefficient is capped at unity at the stall AoA. (*b*) Spanwise AoA distributions at six evenly spaced instants in time during the first half of the linear period, from gust onset (*t* = 0 ms) to approximate maximum force saturation (*t* ≃ 0.05 ms), for the NLC hinged system. The arrow identifies forward chronology as the AoA enters the stall region (shaded grey). Note that the spanwise position has been normalized. (*c*) Spanwise force distributions at the same six instants in time for the NLC hinged system. The arrow identifies forward chronology as the initial force distribution flattens out. Note that the spanwise position has been normalized. (*d*) Normalized centre of pressure *l_F_*/*l*. (*e*) Dynamic fuselage reaction Δ*R*. The departure from the LLC line coincides with the onset of force saturation. (*f*) Resulting fuselage velocity $\dot{z}$. (*g*) Rejection terms for the NLC hinged system. The potential line indicates the maximum achievable rejection (that is necessary to keep the fuselage on a perfectly level trajectory).

We devised a simple approximation for such a curve (figure 4*a*), called here the *nonlinear lift curve* (NLC), which has a totally flat stall plateau of *c_L_* = 1 from 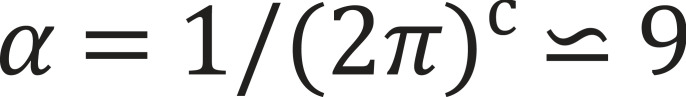 degrees upwards (these values derive from the low-speed sections in Selig *et al*. [26] at bird-scale Reynolds numbers). We then tested the response of the system with this NLC to the same 30% gust (figure 4).

The gust begins to increase the AoA everywhere, as before (figure 4*b*), pushing the lift coefficient towards the stall plateau. This happens first at the root, where the linear upward wing speed is low, and then spreads towards the retreating tip. The inboard force distribution begins to saturate (figure 4*c*). Not only does this limit the resultant force on each wing, but also it keeps the centre of pressure near the centre of percussion for longer, thereby reducing the transmission of reaction to the fuselage (figure 4*d–f*). The saturation effect gradually diminishes as relative down-flow takes over, causing the centre of pressure to drift inboard once more (this is just visible at the wingtip in the later AoA distributions). We also plot the LLC and fixed-wing NLC cases for comparison.

Inertial rejection now precedes the aerodynamic (figure 4*f*). Both curves bear a compelling resemblance to corresponding data in Cheney *et al*. [7] for the owl, whose CFD-derived lift curve (for the whole bird) also exhibits soft-stall behaviour. In fact, many birds' wings stall this way at typical flight Reynolds numbers [27,28] without an abrupt loss of lift. Whether the resemblance between our data and those in Cheney *et al.* [7] is explained entirely by the lift curve is not yet certain, but soft stall definitely enhances the percussion effect in this model and even provides an explanation for the delayed aerodynamic rejection (that the stalled lift on hinged and fixed wings is similar at first). In any case, the lift curve is undoubtedly important to the dynamics of hinged wings.

### 3.4. Increasing the wing mass fraction

**Figure 5.**
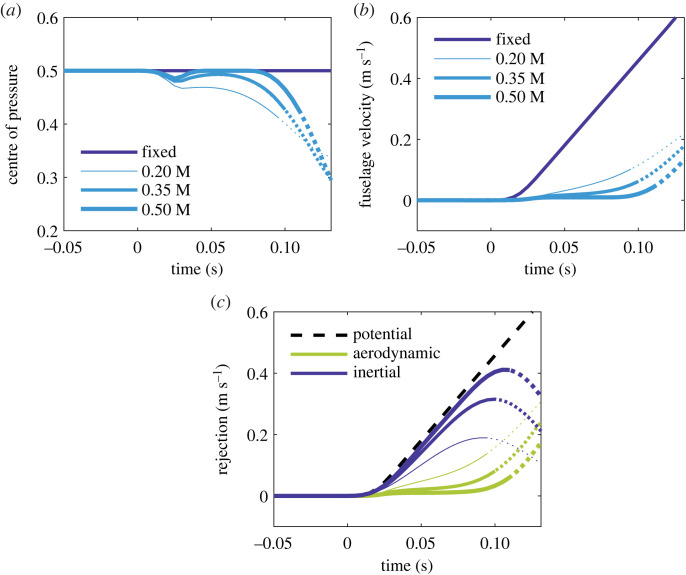
Gust response of the hinged system for three different wing mass fractions, versus the fixed-wing case, with an NLC in the 30% gust. Line thickness denotes the mass of the hinged wing, from 0.2 M to 0.5 M. Plot lines become dotted at the instant the wing angle crosses 20 degrees (approximate onset of nonlinearity). (*a*) Normalized centre of pressure *l_F_*/*l*. Notice that, with increased mass, the wing is slower to exceed the linear threshold; the model therefore captures the initial dynamics even better. (*b*) Resulting fuselage velocity $\dot{z}$. (*c*) Rejection terms for the hinged system. The higher the relative wing mass, the greater the inertial rejection at first.

Each hinged wing has thus far made up 10% of the total system mass (table 1). By increasing the wing mass fraction *μ_w_*, we can prolong and enhance the rejection benefit from soft stall. Figure 5 shows the system dynamics for *μ_w_* = 0.35 and *μ_w_* = 0.5, alongside the existing case (*μ_w_* = 0.2). As the relatively heavy wings present greater resistance to motion, less relative down-flow develops across them when they are gusted. Soft stall therefore happens more readily, and the centre of pressure is stabilized accordingly (figure 5*a*). Indeed, for the wing of highest mass, the lift distribution reaches total saturation; its resultant effectively behaves as unmoving point force, capped in magnitude and fixed at the halfway mark (*l*/2). The fuselage velocity does not change once this begins (figure 5*b*).

Initial aerodynamic rejection falls with increasing *μ_w_* because the fixed and hinged wings each stall quickly with similar total force (figure 5*c*), i.e. their CoM velocities are comparable. Inertial rejection, on the other hand, increases with *μ_w_* and becomes the dominant component.

### 3.5. Hinge torque

Hinge torque determines how well inertial rejection works. Unless the hinge torque is well tuned, the wing will not move as required, no matter where the centre of percussion lies.

Thus far, the hinge torque has been constant and equal to the static value (*T_h_* = *T_h_*_0_). This is the minimum amount necessary to balance the other static moments on the wing and to support flight—i.e. without it, the wings would simply fold up. In the linear model, wings under constant torque respond ‘freely’ to perturbations, experiencing no resistance or assistance to motion during their rotation. The percussion effect can therefore function at its purest, and equation (3.1) applies. However, an aircraft with hinged wings cannot fly under constant torque at all times. The torque must be modulated, probably asymmetrically, to the varied demands of flight, including basic gust recovery (the restoration of the wings to a neutral position) and manoeuvre control.

In this model, hinge torque can be modulated using the dynamic torque parameter Δ*T_h_*, which adds or subtracts from the static value *T_h_*_0_. If retained during the derivation of the original reaction formula, equation (3.1), the dynamic torque gives rise to its own term (see electronic supplementary material part 1, §6), or

3.4 $$\Delta R=\Delta F\frac{\left(P-l_{\Delta F}\right)}{C}+\frac{\Delta T_{h}}{C}.$$

The net effect on fuselage reaction is complicated because the summed terms in this expression interact with one another; dynamic torque affects wing motion (hence aerodynamic load Δ*F*) and vice versa. It is therefore instructive to combine the right-hand side as

3.5 $$\Delta R=\Delta F\frac{\left(\hat{P}-l_{\Delta F}\right)}{C},$$

where $\hat{P}$ is now the ‘dynamic’ centre of percussion,

3.6 $$\hat{P}=P+\frac{\Delta T_{h}}{\Delta F}.$$

Equation (3.5) has the same form as equation (3.1), with $\hat{P}$ in place of *P*. For zero reaction to be maintained as the dynamic torque engages, force Δ*F* would have to track the moving point $\hat{P}$ (which begins at *P* at equilibrium).

**Figure 6.**
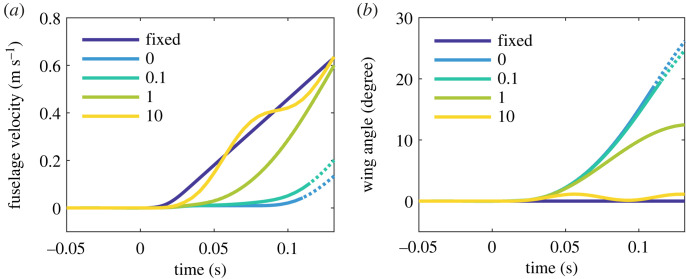
Gust response of the hinged system for three different hinge stiffnesses, versus the fixed-wing case, with an NLC in the 30% gust. Plot lines become dotted at the instant the wing angle crosses 20 degrees (approximate onset of nonlinearity). Dynamic torque comes from a hinge that behaves as a linear torsional spring, for which Δ*T_h_* = *k_t_θ*. The legend gives the stiffness constant *k_t_* (N m rad^−1^) for each case. (*a*) Fuselage velocity $\dot{z}$. (*b*) Wing angle *θ*. For the stiffest hinged system, oscillations begin as the lift coefficient hits the stall plateau. This case is illustrative; no well-tuned inertial rejection system would actually be so stiff or permitted to oscillate in this way.

Consider now the introduction of dynamic torque that mimics the behaviour of a linear torsional spring, or Δ*T_h_* = *k_t_θ*. This simple case will illustrate well the sensitivity of inertial rejection to the mechanical properties of the hinge. Figure 6 shows the effect of various stiffness constants (*k_t_* = 0.1, 1, 10 N m rad^−1^) on the dynamics of the gusted NLC system, alongside the original case (equivalent to *k_t_* = 0 N m rad^−1^), this time for *μ_w_* = 0.5. As the wing enters the gust front and the hinge engages under extra positive (upward) aerodynamic force, the quotient Δ*T_h_*/Δ*F* increases and $\hat{P}$ begins to travel outboard from its starting position at *P*. Zero reaction demands that the centre of pressure of this force follows $\hat{P}$; relative down-flow tends to prevent this, however, and so reaction develops as the two points diverge. The fuselage is thereby pushed upwards (figure 6*a*). Indeed, the higher the stiffness, the quicker this happens, until in the limit of infinite *k_t_* the system responds just as it would with fixed wings. Overall, hinge stiffness therefore impedes wing motion, and with it the capacity for inertial rejection. The same is true for other mechanical elements that develop resistive torque, including dampers.

## 4. Discussion

We show how hinged wings can absorb the initial impact of wing perturbations via the basic mechanics of inertial rejection and the percussion effect. We then propose a method to reject upgusts, which requires: (i) preliminary alignment of the centres of pressure and percussion at equilibrium, i.e. appropriate lift and mass distributions; (ii) hinges that produce constant initial torque; and (iii) wings whose sections stall readily, but softly, onto a lift plateau. Relatively heavy wings are also preferable. Finally, we find that spring-type stiffness at the hinge restricts the motion of the gusted wing, and with it the rejection benefit. Systems with ever stiffer hinges eventually behave as though they had fixed wings (see also [11,29]).

### 4.1. Mechanics of the hinged wing

Successful gust rejection necessitates a hinge mechanism that can supply the necessary static *and* dynamic torques. The specific requirements are: (i) support of the static flight loads; (ii) acceptance of the initial motion of the gusted wing with correctly tuned torque, such that the fuselage is isolated from the disturbance; (iii) gradual arrest of the wing while the gust load is modulated via aerodynamic rejection; and (iv) prompt restoration of the neutral wing configuration without jolting the fuselage. We leave the detailed mechanical implementation of such a hinge for future work, but acknowledge here that the problem is broadly analogous to the design of a suspension system for a terrestrial vehicle. The wings are equivalent to the wheels (the unsprung masses) and the fuselage to the cabin (the sprung mass).

An ideal suspension system provides both *ride quality* and *holding ability*. Ride quality results from isolation of the sprung mass (fuselage or cabin) from the disturbance and requires appreciable deflection of the unsprung masses (wings or wheels). Holding ability concerns handling and stability, and functions better when deflection of the unsprung masses is limited. As such, these are conflicting criteria [30]. Basic passive spring-damper suspension systems cannot resolve this conflict and must usually compromise on both counts (this may exclude them from consideration in the proposed rejection method, for which the hinge must work ‘freely’ at first, then in decisive nonlinear fashion). An adaptive active-passive hybrid or fully active suspension system, on the other hand, could provide a better overall solution. These have been commonplace in automotive applications for decades [31]. Consider, for example, a wing hinge with an active mode that can modify its torque to the instantaneous demands of inertial gust rejection. The hinge would behave passively at first, buying time for the active mode, or, in a *fully* active set-up, be driven by a particularly powerful actuator that could mimic the necessary ‘passive’ dynamics. Other measures, such as control-surface deflection, might then provide follow-up aerodynamic rejection. Active methods also permit real-time tuning of the hinge for other flight objectives [5], including atmospheric energy harvesting, and even allow for adjustments to the dihedral angle(s) for adaptive lateral stability. Ultimately, the designer must decide whether the versatility of hybrid/fully active systems justifies the inevitable mechatronic complexity, extra weight and power demand.

For the system with constant-torque hinges, rejection begins with alignment of the centres of pressure and percussion. This requires suitable distributions of wing lift and mass at equilibrium. On most conventional finite wings, however, the spanwise centre of pressure will naturally lie somewhere near or inside the halfway mark—elliptical loading gives 0.42*l*, for example—and may be difficult to modify without radical alterations to the basic planform or twist geometry. Designers should expect to tune the mass distribution of the wing instead, weighting it towards the hinge for a favourable centre of percussion. The linear mass distribution is a good starting point; it is realistic, given the usual requirement for structural thickness near the wing root, and might even be achieved by clever placement of electronics, fuel, batteries or other onboard items.

### 4.2. Stall aerodynamics

Stall is governed by the evolution of the boundary layer with AoA, which itself depends chiefly on the Reynolds number and the shape of the section(s) that make up the wing [22]. Soft stall, in particular, can be achieved by sculpting a wing section to have a surface pressure distribution that slows down the movement of the boundary-layer transition region with incidence [23,25] for favourable separation behaviour. Many extant wing sections stall softly; Selig *et al*. [26] provide several conventional designs that do so at bird-scale Reynolds numbers (less than 10^5^), while data from Schmitz [32] even show similar behaviour for the simple flat plate. However, the extent to which any of these maintains its soft-stall behaviour during the gust will depend on the timing of the event (the *reduced frequency*) and the associated boundary-layer dynamics. Fast, extreme changes in the flow, including steep gusts, may cause a *dynamic stall* that pushes boundary-layer separation to an AoA some way beyond the usual value, thereby extending the linear region of the lift curve [21]. The curve will then resemble the LLC of §3.2. This is an important, open question that motivates further research on low-speed wing sections for optimal stall, including the potential role of boundary-layer control, e.g. suction, blowing or surface-mounted devices [33].

Wings of relatively high mass (§3.4) have greater rotary inertia and pivot less easily when gusted. The relative down-flow from acquired motion, which acts to oppose the effect of the upgust, is therefore weaker, and soft stall has a better opportunity to develop. If this happens everywhere across the wing, soft stall stabilizes the position of the lift vector and extends the percussion effect, buying even more time for other corrective actions to initialize. The benefit is appreciable; near-zero fuselage reaction lasts for approximately 100 ms in the case of the heaviest wing, which is long enough for a control system to sense and react to the disturbance. Of course, operating at a high equilibrium AoA *α*_0_ (6 degrees here) also facilitates stall.

### 4.3. Avian gust rejection

Birds’ wings, despite their diversity in planform [34,35] and structural complexity, have spanwise mass distributions that are broadly linear, becoming higher nearer the shoulder, with local peaks at the elbow and wrist [16,36,37]. As such, the anatomy naturally bears a mass distribution that puts the centre of percussion near the halfway mark. Now, assuming the lift distribution on these wings is broadly elliptical (exact elliptical lift puts the centre of pressure at 0.42*l*), then close equilibrium alignment between the centres of pressure and percussion may be quite widespread among species—particularly the gliding birds who stand to benefit most from inertial rejection. Of course, the shoulder must be sufficiently compliant, whatever the mass distribution, otherwise the mechanics cannot work at all.

## 5. Conclusion

We present an aeromechanics model of the response of a bird-scale gliding aircraft to a strong, wide upgust. Unlike conventional aircraft, this one has wings that are fully hinged to the fuselage on pin joints that enable rotation in the vertical plane. The hinged design was inspired by the response of birds to upgusts, as measured in a laboratory experiment.

Hinging allows the perturbed wings to absorb and reject the brunt of the gust. The rejection can be optimized by having two key spanwise points on the wing, the centres of *pressure* and the *percussion*, start and stay in good alignment during the early moments of the gust. The initial transmission of load to the fuselage is thereby delayed and/or reduced (which would buy time for other flight control processes to initialize). We call this the ‘percussion effect’. Having presented the basic mechanics, we propose a passive method for achieving the effect in upgusts. The essential ingredients are: (i) appropriate lift and mass distributions for equilibrium alignment of the two key points; (ii) hinges under constant initial torque (enough for aircraft weight support but no more or less); and (iii) a wing whose sections stall softly, such that the centre of pressure is stabilized during gusted rotation.

We ultimately envision the mechanics of the percussion effect as part of a complete hinged-wing suspension system, primarily for small aircraft operating in the gusty conditions of the low atmosphere.

## Online pre-print

A pre-print of an earlier version of this article is available on bioRxiv. https://www.biorxiv.org/content/10.1101/2022.08.05.502962v2.

## Data Availability

The data are provided in the electronic supplementary material [38].
